# A Two-Cycle Audit of Oxygen Prescription in Hospitalised Patients Receiving Supplemental Oxygen: A Retrospective Study

**DOI:** 10.7759/cureus.98732

**Published:** 2025-12-08

**Authors:** Pyae Phyo Thinn, Thet Htar Swe, Mark Delicata

**Affiliations:** 1 Medicine, Victoria Hospital Kirkcaldy, Kirkcaldy, GBR; 2 General Medicine, Victoria Hospital Kirkcaldy, Kirkcaldy, GBR; 3 Care of Elderly and General Medicine, Victoria Hospital Kirkcaldy, Kirkcaldy, GBR

**Keywords:** oxygen audit, oxygen prescription, oxygen qip, supplemental oxygen, target oxygen saturation

## Abstract

Background

Supplemental oxygen therapy is an essential intervention for patients with respiratory insufficiency, but its prescription and documentation are often inconsistent. Oxygen is considered a drug, and improper prescribing or absent documentation can result in inadequate oxygen delivery, potentially leading to harmful effects. Despite national guidelines recommending target oxygen saturation ranges for patients, previous audits, such as the 2015 British Thoracic Society study, indicated significant gaps in oxygen prescription practices across hospitals. The aim of this audit was to assess the accuracy of oxygen prescriptions in hospitalised patients receiving supplemental oxygen, and to evaluate the effectiveness of interventions designed to improve compliance.

Methods

This audit was conducted at Victoria Hospital, Kirkcaldy (NHS Fife), UK, during the first and second weeks of November 2024. A retrospective, cross-sectional study design was used, and data were collected from 69 patients who were receiving supplemental oxygen in the acute medical and general medical wards. Exclusion criteria involved patients under 18 years old, those admitted to specialised units such as A&E, MHDU, or ICU, and patients not receiving supplemental oxygen.

Results

In the first audit cycle, only 19% of the 69 patients receiving supplemental oxygen had an appropriate prescription documented on their medication chart. The majority of patients lacked any formal documentation regarding their oxygen therapy. Following this, two interventions were implemented: a poster campaign to raise awareness about oxygen prescription requirements and a mass email reminder to resident doctors. In the second audit cycle, with a sample size of 77 patients in the same acute and general medical wards, the proportion of patients with a valid oxygen prescription increased to 34%, indicating a positive trend towards improved compliance; it is statistically significant (p-value = 0.04). A p-value <0.05 is considered statistically significant.

Discussion

This audit underscores the ongoing challenge in ensuring adequate oxygen prescription practices in hospitals. Despite clear guidelines and local policies, the initiation of oxygen therapy frequently occurs without a corresponding prescription. The improvement seen after simple educational interventions suggests that targeted strategies can enhance staff compliance, but further efforts are necessary. A notable barrier is the rotation of resident doctors every four months, which disrupts continuity and diminishes the long-term effectiveness of interventions.

Conclusion

While compliance with oxygen prescription among hospitalised patients remains suboptimal, interventions such as awareness campaigns and email reminders have shown some success in improving practices. Ongoing education and regular re-auditing are crucial to ensure continued adherence to best-practice guidelines, ultimately enhancing patient safety and care quality.

## Introduction

Supplemental oxygen therapy is a critical intervention for patients with respiratory insufficiency, yet its prescription and documentation are not always consistent. Proper documentation of oxygen therapy on the medication chart is essential to ensure safe and appropriate delivery, monitoring, and adjustment. Oxygen is a drug; therefore, incorrect or absent prescriptions can result in suboptimal treatment or harm. Inadequate oxygen fails to improve tissue oxygenation, while over-oxygenation can cause adverse effects, such as suppression of hypoxic drive in chronic obstructive pulmonary disease (COPD) and Type 2 respiratory failure patients, barotrauma to lung tissues, and dryness or irritation of the respiratory tract mucosa [1,2].

Oxygen plays a fundamental physiological role. In hypoxaemic states, supplemental oxygen increases arterial oxygen content, thereby improving tissue oxygen delivery and preventing organ dysfunction, cellular injury, and death. In the peri-operative (surgical and post-operative) and acute care settings, hypoxaemia has been associated with increased postoperative complications, delayed wound healing, cognitive dysfunction, and higher mortality. Conversely, emerging evidence indicates that liberal oxygen administration - leading to hyperoxia - is also harmful, being associated with increased oxidative stress, vasoconstriction, lung injury, and worsened clinical outcomes in critically ill and cardiac patients [3,4]. These findings underscore that oxygen therapy is not benign and that target saturation ranges, delivery modes, and monitoring are needed to strike a balance between correcting hypoxia and avoiding hyperoxia.

The British Thoracic Society (BTS) guideline for oxygen use in adults in healthcare and emergency settings recommends that oxygen should be prescribed to achieve a target saturation of 94%-98% for most acutely ill patients, or 88%-92% (or a patient-specific range) for those at risk of hypercapnic respiratory failure [1]. Best practice further recommends prescribing a target saturation range for all hospital patients at admission, to allow prompt and appropriate oxygen therapy if hypoxaemia develops. In the nationwide BTS Emergency Oxygen Audit (2015), 42.5% of patients receiving supplemental oxygen had no valid prescription, despite 70% of hospitals having a policy of setting target saturation ranges [2]. Subsequent audits and reviews highlight continuing deficits in oxygen prescribing, monitoring, and documentation [5-7].

Despite clear national guidelines and local policies, there is a persistent gap between guideline-based practice and real-world prescribing of oxygen. Given this, we conducted an audit at Victoria Hospital, Kirkcaldy (NHS Fife), UK, to assess whether hospitalised patients receiving supplemental oxygen had a corresponding prescription on the medication chart, to implement interventions, and to re-audit to evaluate improvement in documentation and compliance.

## Materials and methods

This audit was conducted at Victoria Hospital, Kirkcaldy (NHS Fife), to assess whether hospitalised patients receiving supplemental oxygen had a corresponding prescription on the medication chart. Data were collected retrospectively from the acute medical and general medical wards during the first and second weeks of November 2024.

This study uses a retrospective cross-sectional audit, with a sample size of 69 patients in the first cycle and 77 patients in the second cycle. Patients were eligible for inclusion if they were admitted under general medicine, were aged 18 years or older, and were receiving supplemental oxygen. Patients were excluded if they were younger than 18 years, admitted to A&E, MHDU, or ICU, or under non-medical specialities and not requiring supplemental oxygen.

Data were collected from medication charts (Kardex), nursing records, and medical records to determine whether a valid oxygen prescription was present (i.e., documented on the medication chart). Simple descriptive statistics (number and percentage) were used. Statistical associations were assessed using IBM SPSS Statistics for Windows, Version 23.0 (Released 2015; IBM Corp., Armonk, NY, USA), using the Chi-square test, with significance defined as a p-value less than 0.05.

## Results

First audit cycle

**Figure 1:**
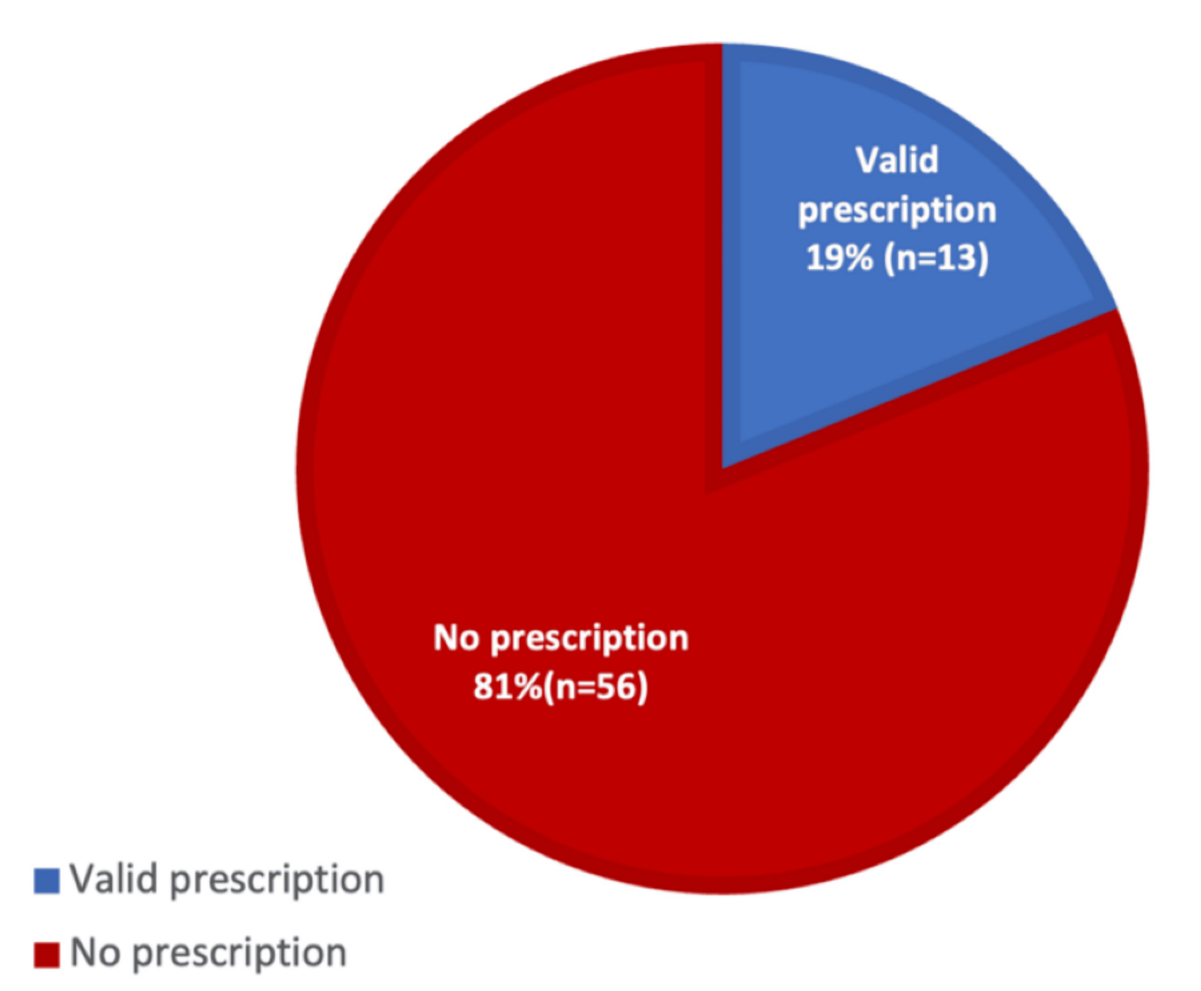
Audit first cycle - percentage of patients on supplemental oxygen with a valid oxygen prescription Data are presented as percentage (%) and number (n) of patients.

Out of 69 patients receiving supplemental oxygen, only 13 (19%) had oxygen therapy prescribed on the medication chart, while 56 (81%) lacked documentation of oxygen prescription (Figure 1).

Interventions

Following the first audit cycle, two interventions were implemented: poster campaign and email reminder.

Poster Campaign

Posters were displayed in doctor rooms and on display boards in the acute medical unit and general medical wards to raise awareness of oxygen prescribing requirements. This initiative was implemented after the first cycle and remained in place throughout the second cycle (see Appendix Figure 3). The poster prominently featured key messages, including reminders of BTS guidelines, target saturation ranges, and proper oxygen-prescribing steps.

Email Reminder

A mass email was sent to resident doctors, encouraging compliance with oxygen prescription protocols (see Appendix Figure 4).

Second audit cycle

**Figure 2:**
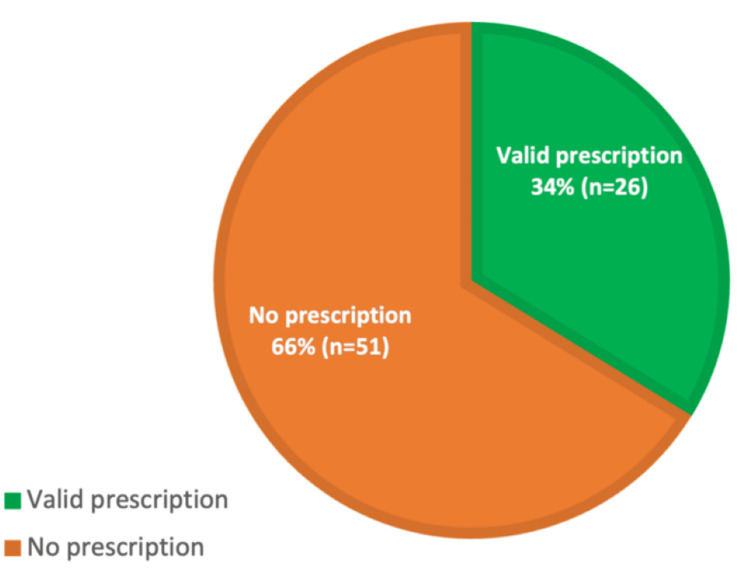
Audit second cycle - percentage of prescription with oxygen in patient receiving supplemental oxygen Data are presented as percentage (%) and number (n). Comparison with the first audit showed a significant improvement (p-value = 0.04). Statistical significance is defined as p-value < 0.05.

A re-audit was performed after these interventions, with a sample size of 77 patients. The proportion of patients with a valid oxygen prescription increased to 26 (34%), compared to 13 (19%) in the first cycle (Figure 2). This represented a positive trend toward improved compliance; the statistical analysis between the first and second cycles, using the Chi-square test, demonstrated that the difference was both statistically significant (p-value = 0.04) and a clinically meaningful change. A p-value < 0.05 was considered statistically significant (Table 1).

**Table 1:** Comparison of valid oxygen prescriptions between first and second audit cycles Data are presented as numbers (n) and percentages (%) and are analysed using IBM SPSS Statistics for Windows, Version 23.0 (Released 2015; IBM Corp., Armonk, NY, USA).

Audit Cycle	Total Patients (n)	Valid Prescription (n, %)	No Valid Prescription (n, %)	Chi-square (χ²)	df	p-value	Statistical Significance
First Cycle	69	13 (19%)	56 (81%)	-	-	-	-
Second Cycle	77	26 (34%)	51 (66%)	4.19	1	0.04	Significant (p < 0.05)

## Discussion

This audit highlights the persistent gap between best practice in oxygen prescribing and clinical practice. Despite clear national guidelines and local policies, oxygen therapy is often initiated without a formal prescription. The improvement observed after simple interventions (poster and email campaign) underscores the effectiveness of educational and behavioural strategies in promoting compliance. However, the rate of proper documentation remains suboptimal, suggesting that repeated and regular interventions are needed [6,8-11].

Our findings are consistent with previously published audits of oxygen prescribing. For instance, the BTS Emergency Oxygen Audit 2015 found that 42.5% of patients on supplemental oxygen had no valid prescription [2]. Single-centre audits at tertiary hospitals in Nottinghamshire and North Wales reported baseline compliance as low as 5%-7%, improving to 50%-88% following multiple PDSA (Plan-Do-Study-Act) cycles [8,12,13]. Other audits reported 45% compliance at Salford Royal NHS Foundation Trust [4] and 54.8% at Royal Blackburn Teaching Hospital [14]. Nevertheless, all these rates remain below the BTS target of 95% in the 2015 national guideline [2]. Achieving the 95% target may be challenging in practice due to factors such as the complexity of patient management, time constraints in acute settings, and variability in staff adherence to guidelines. Moreover, in the context of current NHS settings, where resources and staffing pressures are often significant, it may be more realistic to aim for continuous, incremental improvements in compliance rather than expecting full adherence across all settings.

Compared with these, our baseline compliance (19%) is higher than in some single-centre reports, but still well below the standard of full adherence, and our improvement (to ~34%) is in keeping with the magnitude of gains seen elsewhere following educational interventions, but remains short of the >50% compliance achieved in centres with more intensive, system-level efforts [9,10].

A key barrier to maintaining the long-term effectiveness of our intervention is the four-monthly rotation of resident doctors, which limits the impact of educational interventions and disrupts sustained good practice. The continued use of FEWS (Fife Early Warning System score) instead of NEWS2 (National Early Warning Score 2), alongside paper-based oxygen prescription practices, appears to underestimate the need for supplemental oxygen in some patients. Other barriers include a lack of integrated prescribing prompts in electronic medical records, time pressures during ward rounds, and the staff perception that oxygen does not require a formal prescription [5,11].

Possible new measures to improve oxygen prescription include the introduction of oxygen prescription checks during ward round checklists, education of rotating doctors during induction programs, regular training of healthcare professionals, and empowering respiratory nurses or pharmacists to ensure ongoing compliance [11].

A recent study described an automated audit of hospital oxygen use, demonstrating the potential for continuous monitoring and feedback when integrated into electronic health records [7]. If this occurs in our NHS trust, it will subsequently improve our oxygen prescription ratios. Our NHS trust is also trying to adopt the new NEWS2, which offers important advantages over many local early warning systems when it comes to oxygen therapy. First, it incorporates a specific oxygen saturation scale for patients with, or at risk of, hypercapnic respiratory failure (target SpO₂ 88%-92%), which helps tailor oxygen prescription and minimise the risk of over-oxygenation in vulnerable groups. Second, it includes a prompt for supplemental oxygen (a binary “on oxygen” parameter) as part of its scoring algorithm, thereby linking changes in oxygen requirement directly to a higher alert level - this can aid early review and adjustment of therapy. Finally, because NEWS2 is nationally standardised, it fosters consistency across wards and hospitals, reducing variation in oxygen prescribing practice that may occur with locally defined systems [15,16].

Future audits should broaden their scope to evaluate not only the presence of a prescription, but also adherence to target saturation ranges, documented flow rates, duration of oxygen therapy, actual patient saturations achieved, and patient outcomes (e.g., incidence of hypercapnia and length of stay) [6,13].

Limitations

The study had several limitations. Staff rotation may have affected the consistency of data collection and follow-up, as audit team members were resident doctors rotating through different departments. Data were collected only during specific weeks, and patient turnover or oxygen prescription practices may have varied at other times. The study included a relatively small number of patients from a single hospital, which limits generalisability. Some charts may have contained incomplete or ambiguous documentation, potentially affecting data accuracy. Finally, the re-audit occurred soon after the interventions, possibly not allowing enough time for behavioural changes to be fully adopted [9].

## Conclusions

Oxygen prescription compliance among hospitalised patients receiving supplemental oxygen remains low but showed measurable improvement after simple interventions. Continued efforts, focusing on awareness, education, and system-based reminders, are necessary to achieve full adherence to best-practice guidelines. Rolling re-auditing will help ensure sustained improvement in patient safety, care quality, and clinical outcomes.

## Figures and Tables

**Figure 3:**
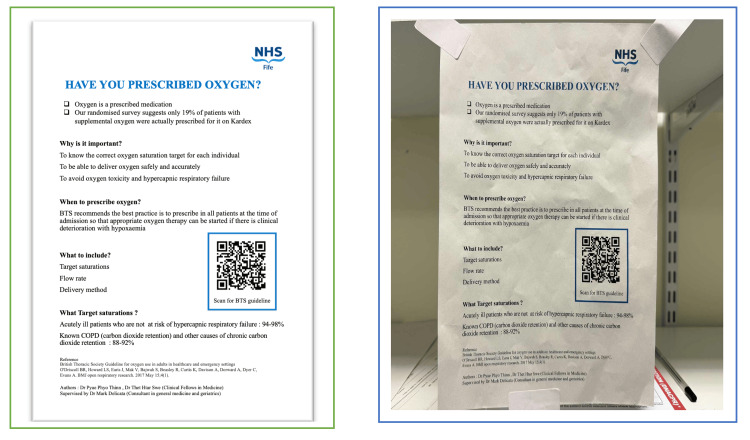
Intervention: poster campaign

**Figure 4:**
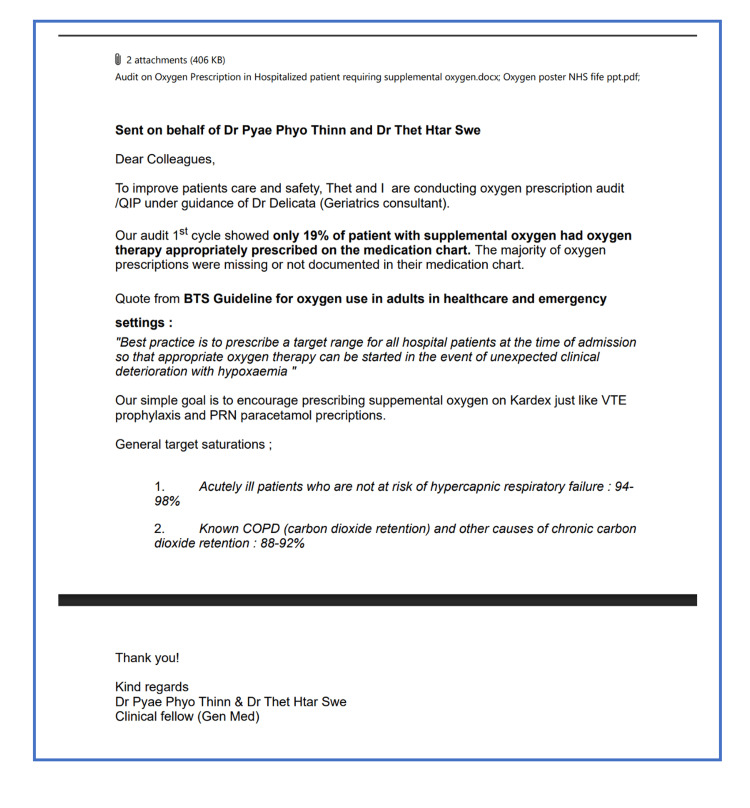
Intervention: raising awareness via mass email
